# Myth busting? Effects of embryo positioning and egg turning on hatching success in the water snake *Natrix maura*

**DOI:** 10.1038/srep13385

**Published:** 2015-08-21

**Authors:** Fabien Aubret, Gaëlle Blanvillain, Philippe J. R. Kok

**Affiliations:** 1 Station d’Ecologie Expérimentale de Moulis, CNRS, 09200 Moulis, France; 2 Biology Department, Amphibian Evolution Lab, Vrije Universiteit Brussel, 2 Pleinlaan, B-1050 Brussels, Belgium

## Abstract

It is a common belief that reptile eggs should not be turned after oviposition once the embryo has attached itself to the inner membrane of the shell as it might kill developing embryos. Here, we used 338 eggs from 32 clutches of the water snake *Natrix maura* to (1) thoroughly describe natural clutch arrangement, (2) experimentally assess the effects of natural embryo positioning and (3) egg turning on embryo metabolism, hatching success, and hatchling phenotype. Clutches contained, on average, 59% of embryos located at the top, 28% at the bottom, and 14% on a side of the egg. Larger females laid larger clutches with higher proportion of top located embryos. Top embryos displayed higher metabolic rates (heart rates), shorter incubation time, and produced lighter and shorter snakes than bottom embryos. Egg turning did not significantly influence egg development, hatching success or hatchling phenotypes. However, post-birth mortality was significantly higher in turned (37.5%) compared to unturned (4.5%) embryos, providing support to the common belief that eggs should not be moved from their natural position.

The physical conditions experienced during incubation and the consequence for hatching success, hatchling phenotypes and overall fitness have been studied in a wide range of taxa, from birds, reptiles, fish, amphibians, to insects^1^,^2^,^3^,^4^,^5^. Incubation temperature, and in relevant taxa, hygrometry, have dominated most of the research effort and proven to be paramount drivers of hatching success and hatchling phenotype^6^,^7^,^8^,^9^,^10^,^11^,^12^. Nevertheless, the roles played by more subtle factors such as the relative position of individual eggs within the clutch^13^,^14^, or the position of the embryo within the egg during incubation has attracted less attention, perhaps due to technical difficulties^4^. Yet, driven by the poultry industry research and development, the role and benefits of egg rolling in birds has been extensively studied^15^,^16^,^17^. Bird eggs benefit from turning because it facilitates embryonic development by stimulating the growth of the area vasculosa, which in turn maximizes nutrient uptake from the yolk^4^.

Contrary to bird eggs, it is generally assumed that reptile eggs do not respond well if rolled or turned early on or after laying^18^. It is indeed common “knowledge” amongst amateur and professional reptile breeders that freshly laid eggs should be marked with a pencil to distinguish top from bottom prior to being placed in an incubator (i.e. in the exact same position as they were found). This belief most likely stems from the observation that reptilian eggs lack the avian chalazae^19^,^20^. In the absence of specific experimental studies, whether this belief belongs to the “urban myth” category or is indeed true remains an open question. Nevertheless, it is assumed that within a few hours after oviposition, reptile embryos rise to the top of the egg and start adhering to the inner membrane of the shell^18^,^21^. Thus, if the egg is turned after the embryo has attached itself, the weight of the yolk could impede normal development or tear both the vitelline and extra-embryonic membranes^18^,^22^,^23^, leading to death or malformations^18^,^21^,^22^,^23^.

Experimental observations about the effects of egg handling or turning in reptiles are scarce, and often anecdotic. No effect on egg development and hatching success were reported in the garden lizard *Calotes versicolor*^24^, leopard geckos *Eublepharis macularius*^25^, most freshwater turtles^21^,^26^,^27^,^28^,^29^, and, to some extent, in the alligator *Alligator mississippiensis*^30^. In other taxonomic groups, deleterious effects have been reported, for example in the desert iguana *Dipsosaurus dorsalis*^31^ and in most sea turtles species^32^,^33^,^34^. However, experimental designs and testing techniques varied greatly across studies, making any trends difficult to characterize. Snakes appear to be the least studied group in this regard. Pawley^35^ observed no effect of “daily rotation of some *Natrix natrix* eggs”, and Marcellini and Davis^25^ reported that egg turning did not affect hatching success in *Pantherophis guttatus* (results for *Python molurus* were unclear). With a few exceptions (i.e. pythons, cobras^36^), snakes do not care for their egg (apart from selecting for nest-site^37^), and usually leave the nesting site within hours post**-**laying^37^,^38^. Hence, eggs may incubate in the position they were laid or arranged by the female. Snake clutches are usually clustered, with eggs strongly attached to each other. Within minutes of laying, eggs often become difficult to separate without tearing the soft shell^39^,^40^. Why snake eggs are attached to each other is unknown. An obvious possibility is that egg clustering/attachment may prevent the eggs from rolling during incubation, therefore implying that the maintenance of embryo positioning within the egg volume once attached to the egg membrane is an important factor to hatching success and/or hatchling phenotypes and survival.

Here we tested this hypothesis using eggs of the water snake *Natrix maura*. We first collected detailed information of embryo positioning and potential embryo movements within the egg volume following egg laying, and incubated the eggs in the exact position they were found. In a second experiment, we purposely manipulated and incubated freshly laid eggs with top embryos by turning half of them into a down position. We thoroughly assessed the potential effects of embryo positioning and egg turning on egg development (egg mass trajectories, heart rates), hatching success, and hatchling phenotypes (body size at birth, growth rates, heart rates, behavior, and locomotor performances).

## Results

### Experiment 1: Clutches arrangement and embryo location

**Figure 1:**
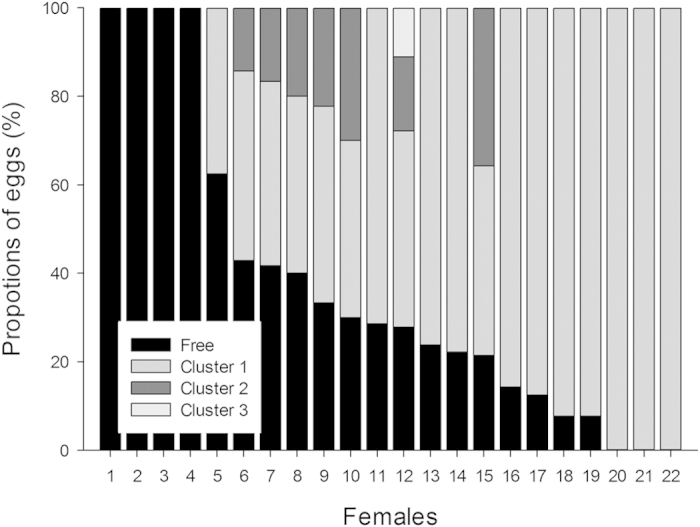
Egg arrangement, described in 22 clutches of the water snake *Natrix maura*, varied significantly across clutches (*χ*^2^_22_  = 120.28, P = 0.0001), ranging from 100% free eggs (i.e. no eggs were attached to each other) to 100% clustered eggs (in one, two or three distinct clusters).

Egg arrangement varied significantly across clutches (*χ*^2^_22_ = 120.28, P = 0.0001), ranging from 100% free eggs (i.e. no eggs were attached to each other) to 100% clustered eggs (in one, two or three distinct clusters—Fig. 1). Egg attachment occurred in 18 out of 22 clutches for which 76.9 ± 17.2% of the eggs within the clutch were attached. Larger clutches contained more attached eggs (Linear regression *R* = 0.88, *F*_1, 16_ = 54.55, P = 0.001).

The proportion of eggs with top (embryo located at the top of the egg), bottom (embryo located at the bottom) or side (embryo located on a side) embryos were significantly different between free and attached eggs (Table 1; Pearson *χ*^2^_2_ = 20.01; P = 0.0001). Clustered eggs had a higher proportion of top embryos than free eggs (respectively 67.7% *versus* 43.3%; *χ*^2^_1_ = 19.96; P = 0.0001). Overall, 58.6% of eggs had top embryos, 27.6% bottom embryos, and 13.8% side embryos.

**Figure 2:**
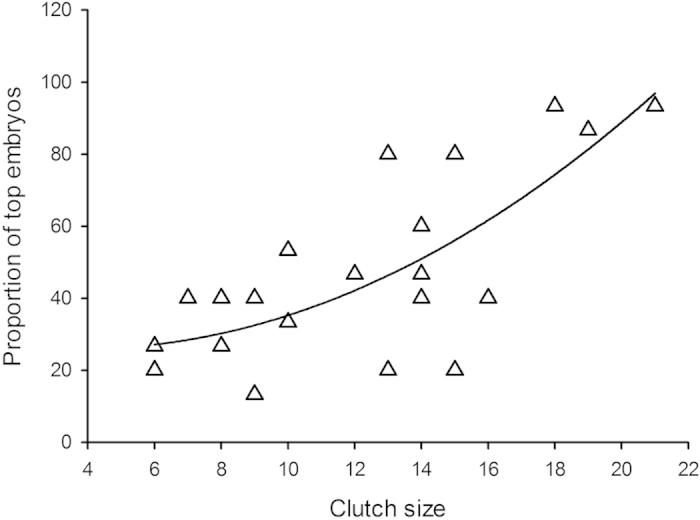
A total of 263 eggs (N = 22 clutches) of water snake were checked for the positon of their embryos within 12 hours post-laying. The proportions of eggs with top embryos (i.e. embryos located at the top of the egg) increased as non-linear function of clutch-size. Regression line is plotted as a quadratic fit (r = 0.75; F_2, 19_ = 12.27; P = 0.0004; equation *y* = −1.7925*x* + 0.2385*x*^2^ + 29.3186).

A multiple regression analysis with mother snout vent-length (SVL) as continuous predictor and the number of top embryos, the number of attached eggs, and clutch size as dependent variables yielded Wilks’ λ = 0.51; F_3, 18_ = 5.71; P = 0.006. Mother SVL significantly explained the variation in the proportion of eggs with top embryos within a clutch, as well as clutch size, but not the variation of eggs attached to each other in a clutch (Table 2). Further, the proportion of eggs with top embryos increased as a non-linear function of clutch-size (see Fig. 2).

Embryo drifting up within 24 hours post-laying was minimal and observed in only 13 out of 90 eggs, averaging 4.2 ± 2.2 mm (*Natrix maura* eggs average 26.9 ± 2.3 mm in length by 16.6 ± 1.6 mm in width, N = 574, unpublished data). No embryo relocation (i.e. from top to bottom for example) was observed after up to five days post-laying. When both attached and free eggs were present in a clutch (N = 18 clutches), they did not significantly differ in mass at laying (two-way Anova with female and egg status as factors; effect of status F_1, 158_ = 0.15, P = 0.70; interaction term F_14, 158_ = 0.65, P = 0.82).

Because side embryos could not be clearly assigned to the top or bottom category, and because some clutches lacked side embryos, we discarded side embryos from this analysis, and focused on top and bottom positioning only, in order to increase analytical power. The proportion of top and bottom embryos was highly variable amongst clutches (and close to the conventional level of significance; *χ*^*2*^_13_ = 20.67; P = 0.079). Due to the unbalanced design and the lack of significant interaction between the factors litter of origin and embryo position for any of the traits tested, we used two-way factorial Type II Anovas throughout the analysis.

Hatching success was 92.2% for top embryos and 81.8% for bottom embryos (*χ*^2^_1_ = 2.56; P = 0.11). Post-hatching survival (during the first three weeks of life) was similar between the two groups (88.7% *versus* 92.6% for top and down embryos, respectively; *χ*^2^_1_ = 0.32; P = 0.57).

**Figure 3:**
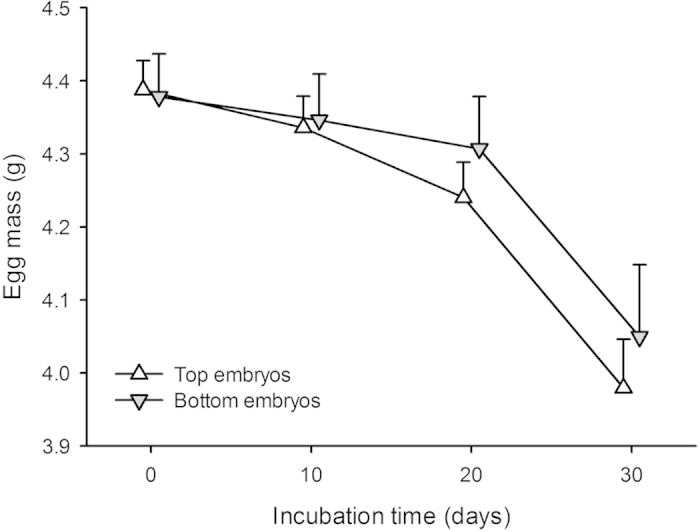
Eggs laid with embryos located at the top (N = 77) lost more mass towards throughout the incubation period than eggs laid with embryos located at the bottom (N = 32). A repeated measure factorial Anova with litter of origin and embryo position as factors, and egg mass as repeated measures over time yielded: global interaction term F_39, 243_ = 1.12; P = 0.30; effect of embryo location across time F_3, 243_ = 3.31; P = 0.021). Means + SE are given.

Incubation time, body mass and snout-vent length at birth were significantly different between top and bottom embryos (see Table 3 for statistical results). There was a significant difference in egg mass evolution throughout the incubation period between top and bottom embryos: the latter lost significantly more mass throughout the incubation than the former (Fig. 3). Post-birth growth rates (without food) in body mass and snout-vent length did not significantly differ between the two groups (repeated measure factorial Anova; global interaction terms both P > 0.36; effects of embryo location both P < 0.017): young snakes born from bottom embryos tended to remain both heavier and longer in the first 3 weeks of life.

**Figure 4:**
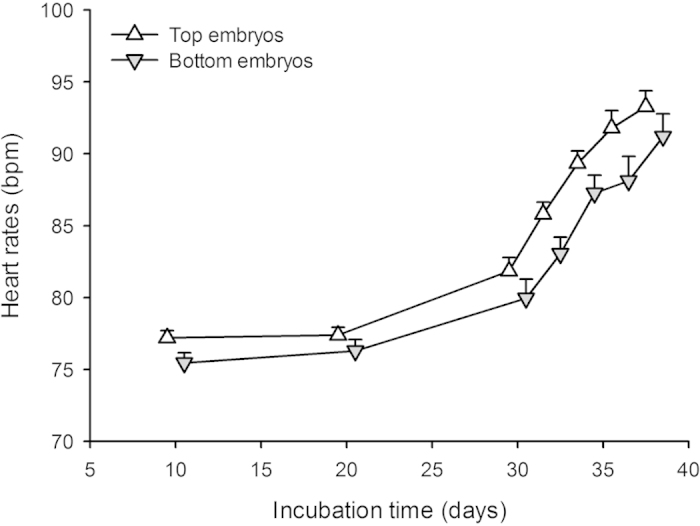
A total of 110 eggs (N = 14 clutches) of the water snake were incubated in the exact position they were laid, including 77 top embryos (located at the top of the egg) and 33 bottom embryos (located at the bottom) and eggs regularly surveyed for their heart rates (i.e. metabolic rate). Heart rates were consistently higher throughout the incubation period in top (85.23 ± 3.33 bpm) compared to bottom embryos (83.04 ± 3.17 bpm—repeated measures Anova; interaction term F_54, 324_ = 1.14; P = 0.24; effect of embryo location F_1, 54_ = 7.29; P = 0.009). Means + SE are given.

**Figure 5:**
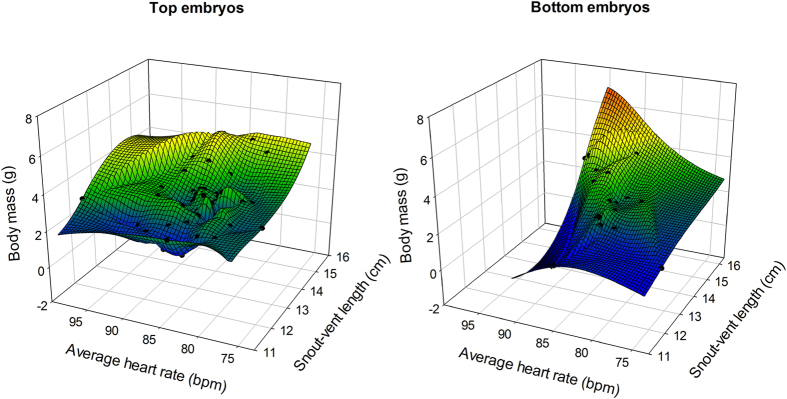
3D plot of mean embryo heart rates recorded throughout incubation plotted against hatchling snout-vent length against hatchling body mass in top (left panel) and bottom (right panel) embryos. Hatchling snout-vent length and body mass were largely explained and positively correlated with average embryo heart rates during incubation in bottom embryos. Such relationship however, was much less pronounced in eggs where the embryos were located at the top of the egg (see text for statistical results).

Heart rates were consistently higher throughout the incubation period in top (85.23 ± 3.33 bpm) compared to bottom embryos (83.04 ± 3.17 bpm—see Fig. 4). A General Linear Model with (1) mean heart rate as a continuous predictor, (2) snout-vent length and body mass at birth as dependent variables, and (3) embryo positioning and clutch of origin as categorical predictors yielded a marginally non-significant effect of embryo positioning F_2, 38_ = 3.11; P = 0.056—see Fig. 5). The difference in heart rates between top and bottom embryos significantly explained the variation in body mass and snout-vent length at birth (see Table 4).

### Experiment 2: manipulation of embryo location and effect of egg turning

We used a split clutch design that balanced the proportion of top and bottom embryos across clutches: *χ*^2^_9_ = 1.80; P = 0.99, hence on way Type I Anovas were used to compare traits between unturned and turned eggs.

There was no significant difference in hatching success between turned and unturned embryos (75.8% *versus* 72.7%, respectively; *χ*^2^_1_ = 0.07; P = 0.80). However post-birth mortality was significantly higher in turned compared to unturned embryos (37.5% *versus* 4.5% respectively; *χ*^2^_1_ = 6.69, P = 0.0097). Birth defect rates were similar between treatments (*χ*^2^_1_ = 0.71, P = 0.40; birth abnormalities were recorded in 2 out of 17 young snakes in turned eggs *versus* 1 out of 22 in unturned eggs). Amongst all traits measured, only emergence time was close to the conventional level of significance (see Table 5).

## Discussion

This study first aimed at providing a thorough description of egg arrangement and embryo positioning in clutches of the water snake *Natrix maura*. As in the vast majority of snake species, *Natrix maura* lay eggs that are firmly attached to each other (most clutches: 18 out of 22) in proportions (77% on average; Fig. 1) that are a direct consequence of clutch-size. When eggs were attached to each other, embryos were mostly located towards the top of the eggs (67.7%) and seemed to retain that position for as long as we could see through the egg membrane (i.e. no significant drifting was observed, as is thought to occur in turtles, lizards and alligators^18^,^21^). The proportion of eggs with top embryos was lower (43.3%) in free eggs however. This suggests that some eggs were accidentally rolled by the female before they had become firmly enough attached to other eggs. Alternatively, some eggs may have remained free because they were purposely laid beside the rest of the clutch by the ovipositioning snake.

Further studies are needed to ascertain (1) the existence and/or timing of embryo migration in snake eggs and (2) that egg positioning occurs in a similar way in natural nests (i.e. less stress and more space available to the females). The use of imagery in gravid snakes for instance, and a close follow up of eggs immediately after oviposition might help determine embryo location prior to and following egg**-**laying.

A second aim of this study was to understand the effect of natural egg positioning on hatching success and hatchling phenotype. Top embryos did not survive better than bottom embryos, and hatchlings from both categories survived equally well. Top embryo eggs however, lost more mass throughout the incubation, hatched earlier and produced lighter and shorter snakes at birth than bottom embryo eggs. This result suggests differential utilisation of yolk, either as a cause or a consequence of differing heart rates (i.e. metabolic rates) throughout the incubation period: top embryos displayed mean heart rates of 85.23 ± 3.33 bpm compared to 83.04 ± 3.17 bpm in bottom embryos.

The final aim of this study was to investigate the effect of purposely disturbing embryo positioning, by inverting naturally positioned eggs with top embryos. Our results do not support the common belief that egg turning in reptiles can kill, or even affect the developing embryo, at least when eggs are turned within 12 hours post**-**laying (as shown in desert iguanas^31^, loggerhead^32^ and green sea turtles^33^). Egg mortality was indeed unaffected by the treatment (as reported in the garden lizard *Calotes versicolor*^24^; and leopard geckos *Eublepharis macularius*^25^). To our knowledge, only one study has carefully looked at the effect of egg handling in snakes^25^. Although sample sizes were rather small (less than 20 eggs per group), the authors reported that egg turning did not affect hatching success in the corn snake *Pantherophis guttatus*. In our study, while hatching success was unaffected, young *Natrix maura* hatchlings born from turned eggs died at much higher rates than snakes born from unturned eggs (37.5% *versus* 4.5% mortality), perhaps from undetected internal malformations. This result was not due to one low quality clutch, as snakes that died originated from 4 different clutches. Moreover, because these juveniles died prior to body size re**-**measurement as well as performance and behavioural testing, the analysis did not include their scores, potentially minimizing the statistical effect of treatments (most trait comparisons yielded non-significant results).

Although further study may assess the effect of egg turning at a later stage in the incubation process, such a study would not be biologically meaningful: the vast majority of snake eggs become strongly attached to each other shortly after being laid^39^,^40^ so that the initial egg position remains the same until hatching. Our results suggest that differential post-birth survival rates of turned *versus* unturned eggs may have favored the evolution of egg-clustering (i.e. eggs attached to each other) in snakes. This prompts the question: would non-attached snake eggs be at risk of being rolled after they have been lai? Although communal nesting is chiefly observed in snakes (only 3% of oviparous species do^41^), it is very common in natricine snakes. Water snakes and grass snakes (*Natrix natrix*) are often found in communal nesting sites^42^,^43^,^44^. Because the timing of egg laying may vary across species and individuals^45^, eggs may be accidentally displaced due to high traffic of reproductive females, refuging snakes, or other types of animals. Egg attachment would primarily prevent this from happening, especially in the first critical hours post-laying. There might be additional benefits to egg clustering in snakes, especially in a natural nesting site (i.e. more prone to attacks from bacteria, parasites, mold, insects) rather than optimal laboratory incubation conditions. By increasing air flow around the eggs, egg clustering may also enhance egg water exchange^46^,^47^,^48^, reduce the risk of mold^49^ or promote hatching synchrony (i.e. egg communication^50^,^51^).

In conclusion, our data suggest that egg turning can be deleterious to the survival of hatchlings and that eggs should be incubated in the position they were found for optimal results (i.e. high hatching success and potential expression of phenotypic syndromes). Further studies are required to better understand (1) the effect of turning bottom embryo eggs into top embryo eggs and (2) the overall benefits of egg clustering in snakes.

## Methods

### Study animals, study site and experiment design

The water water snake (*N. maura*) occurs in France, Spain, Portugal, south**-**west Switzerland, north**-**west Italy and on a few Mediterranean islands^52^. This species is largely aquatic and individuals are always found in the vicinity of water^45^. A total of 32 gravid females were hand-caught in June and July 2013 (N = 22) and 2014 (N = 10) along the banks of the Lez river and surrounding pasture and woodlands in south**-**west Ariège, France; between the localities of Moulis (42° 57′ 43″ N; 1° 05′ 30″ E) and Eylie (42° 49′ 59″ N 0° 56′ 15″ E). They were brought back to the laboratory (CNRS, Moulis) and housed individually in plastic containers containing an egg laying box (see Aubret *et al.*^53^ for details on housing conditions). All experimental protocols were approved by the Préfecture de l’Ariège, which provided capture, breeding, experimentation, release and ethics permits (Arrété #2012**-**11). All experiments were carried out in accordance with the approved guidelines. All females were returned to their exact site of capture within two weeks of egg**-**laying.

#### Experiment 1: clutch arrangement and embryo location

A total of 263 eggs from 22 clutches (mean clutch size = 12.2 ± 0.9) were collected from July 9^th^ to August 14^th^ 2013. Eggs were collected within 12 hours of laying. They were gently detached from their clutch when attached to other eggs, and candled in order to determine embryo position. Developing embryos appear as an obvious red patch along with circling blood vessels contrasting with the white egg shell. Embryo positions were categorized as top (embryo located at the top of the egg), bottom (embryo located at the bottom), or side (embryo located on the side). We also circled developing embryos with a pencil in order to quantitatively track potential drifting of the embryo in the first 24 hours (90 eggs from 6 clutches). Eggs were individually marked using a pencil with a letter (coding for litter of origin) and a number (egg number within each litter) for identification purposes. Eggs were placed in a plastic container (one clutch per container; 20 cm × 15 cm × 5 cm) on a 2 cm layer of vermiculite in the exact position they were found, and were then transferred into an Aqualytic® incubation chamber set at a constant 28 °C. The general embryo location (top, bottom, or side) was rechecked after 24 to 48 hours (N = 19 clutches, 214 eggs total), after 4 days (N = 1 clutch of 11 eggs), and 5 days of development (N = 2 clutches of 8 and 5 eggs, respectively). After 5 days, the egg membrane becomes more opaque, rendering embryo locating much less precise and in most cases impossible.

We incubated 110 eggs from 14 clutches in the exact position they were laid (77 top embryos and 33 with bottom embryos) for this experiment (the remaining eggs were involved in other experiments).

#### Experiment 2: manipulation of embryo location

We obtained 75 eggs from 10 clutches (mean clutch size = 7.5 ± 2.2) between July 29^rd^ and August 24^th^ 2014. To assess the effect of embryo positioning and egg turning on development and survival, we kept only the 51 eggs with top embryos (on average 5.1 ± 2.4 eggs per clutch). Within each clutch, eggs were allocated to two treatments using a split clutch design: eggs were numbered from heaviest to lightest and evenly distributed into two half-clutches. This ensured consistency in egg mass across treatments. Then, all the eggs from one of the half-clutches were inverted (i.e. becoming eggs with bottom embryos; N = 22) while the remaining eggs were left in their original position (i.e. with embryos at the top; N = 29). All eggs were then placed in plastic containers (one clutch per container), and transferred into the incubation chamber set at a constant 28 °C for the duration of the incubation period.

### Egg and hatchling measurements

Eggs were measured in mass to the nearest 0.01 g using a digital scale within 12 hours of oviposition, and then every 10 days throughout the incubation period. Upon hatching, we recorded the delay between egg pipping (slicing of the egg shell by the snake’s birth tooth) and full snake emergence from the egg (hereafter called emergence time). Hatchlings were sexed by eversion of the hemipenes, and marked by scale**-**clipping for identification. All were measured within 12 hours of emergence in body mass (±0.01 g), snout**-**vent length (±0.1 mm) and total length (±0.1 mm). We also recorded obvious (i.e. external) birth defects when present (such as scale abnormalities, deformed tail, microphtalmia, macrophtalmia or micrognathia). The remaining egg shells (along with fluid and unabsorbed yolk) were also weighed (±0.1 g). Siblings were housed together in plastic boxes (15 cm × 10 cm × 5 cm) with a water dish, shelter and paper towel as substrate. Snakes were remeasured in body size at three weeks old. A Body Condition Index (BCI^57^) was calculated for each snake, using the residual values of the linear least**-**squares regression of Log (birth body mass) against Log (birth snout**-**vent length). A residual yolk index (RYI) was calculated as the residual values of the linear least**-**squares regression of Log (egg mass post hatching) against Log (egg mass prior to hatching). All tests were performed on all hatchlings.

### Heart rate measurements

We measured embryo heart rates as well as hatchling heart rates (body temperature 28 °C) using the Buddy® digital egg monitor (MK2, Avitronics) under the protocol described for eggs^54^ and neonate snakes^55^. Embryo heart rates were measured at incubation day 10, 20 and 30, and then every two days until hatching in 2013. In 2014, heart rates were measured first at day 7, and then every 7 days until hatching.

### Performances and behaviour

Tests started when snakes were three weeks old, using a balanced order testing design to take into account potential effects of age and prior experience on the outcomes (see Aubret *et al.*^53^ for details on method). Individual snakes underwent one test a day, allowing a 24 hour rest between tests, so that the whole procedure lasted 5 days.

#### Swimming performance

We used a standard procedure adopted in previous studies^53^. A high definition wide angle digital camera was fitted above a linear 300 cm × 40 cm × 50 cm swimming track and used to record trials (recording section of approximately 120 cm). The tank was filled with 10 cm of water adjusted to 25 °C using a reverse-cycle water chiller (TECO® TC15). The video was then edited on a computer and swimming speed calculated over the first five lengths of the track swum by each snake. The fastest performance was retained for each of the five lengths swum, and a fastest overall performance for the entire swimming test (the first length was usually the fastest).

#### Defensive behavior

Scoring of defensive behavior followed a previously published procedure^56^. Snakes were tested at room temperature (approximately 20 °C). Defensive behavior ranged from (1) body positioning as an S-shape, (2) flattening of the head and/or body, (3) hissing, (4) cobra-like posture (raising of the anterior body and head, ready to strike), to (5) striking at the threat (always with the mouth closed). Each behavior equaled one point. Individual scoring thus ranged from 0 (no reaction) to 5 (full anti-predatory panel displayed).

#### Exploratory behavior

Snakes were individually placed in a small plastic tube closed at one end. The tube containing the snake was then placed in a large plastic box (55 cm × 35 cm × 26 cm), and was left undisturbed for up to an hour, allowing the snake to come out of the tube and explore its surrounding area. Using a digital chronometer, we recorded the time taken by each snake to come out of the tube (that is, at least one third of the body out). Snakes that remained within the tube longer than the set time of 60 minutes were given this maximum score.

Once all tests were completed, snakes were given their first meal (small dead minnows ranging from 0.5 g to 1 g; supplied by Armorvif®) prior to being released at the maternal capture site.

### Data analysis

Assumptions for normality of the data and equality of variances were tested on all variables (Lilliefors and Levene’s tests). Where not normal, requirements were met by Log-transforming the relevant data prior to analysis. Swimming and crawling speed were analyzed in absolute terms (in cm per s) and relatively to snake’s total length (in body length travelled per s). Means ± standard deviations are given unless otherwise stated.

## Additional Information

**How to cite this article**: Aubret, F. *et al.* Myth busting? Effects of embryo positioning and egg turning on hatching success in the water snake *Natrix maura. Sci. Rep.* **5**, 13385; doi: 10.1038/srep13385 (2015).

## Figures and Tables

**Table 1:** Clutch arrangement and embryo positioning in the water snake *Natrix maura*.

Embryo location	Top	Side	Bottom
Attached egg (N)	111	23	30
(*% per clutch*)	*66.20* ± *26.65*	*11.88* ± *14.65*	*21.92* ± *22.69*
Free egg (N)	42	13	42
(*% per clutch*)	*29.76* ± *25.60*	*12.02* ± *21.14*	*58.21* ± *26.62*

**Table 2:** Statistical results of a multiple regression analysis between mother snout vent-length (continuous predictor) and the number of top embryos, the number of attached eggs, and clutch size (dependent variables).

	r	df, F	P
Number of eggs attached	0.41	1, 20; 4.08	0.057
Number of top embryos	0.52	1, 20; 7.25	0.014
Clutch size	0.69	1, 20; 18.71	0.001

**Table 3:** Comparison of traits between snake hatchlings born from embryos located either at the top or bottom of the egg.

Embryo location	Top	Bottom	df, F	P
Incubation duration (days)	44.02 ± 1.49	44.55 ± 1.31	1, 56; 7.36	**0.009**
Emergence time (days)	0.47 ± 0.68	0.61 ± 0.60	1, 56; 1.38	0.25
Residual yolk index	0.024 ± 0.0007	−0.103 ± 0.016	1, 59; 1.78	0.19
Body mass (g)	2.49 ± 0.62	2.66 ± 0.55	1, 56; 4.50	**0.038**
Snout-vent length (cm)	13.07 ± 1.15	13.52 ± 1.01	1, 56; 9.79	**0.003**
Body condition index	−0.00042 ± 0.080	−0.016 ± 0.071	1, 56; 0.81	0.37
Swimming speed (cm.s^-1^)	44.01 ± 9.20	46.79 ± 9.46	1, 45; 0.96	0.33
Swimming speed (body length.s^-1^)	2.64 ± 0.52	2.65 ± 0.54	1, 45; 0.10	0.76
Time to exit shelter (min)	24.27 ± 5.05	30.53 ± 7.49	1, 46; 2.09	0.15
Defensive score	1.48 ± 1.82	2.58 ± 1.83	1, 45; 0.93	0.34

**Table 4:** Statistical results from a General Linear Model between mean heart rate (continuous predictor), embryo positioning and clutch of origin (categorical predictors), and snout-vent length and body mass at birth (dependent variables).

	r	df, F	P
Body mass	0.90	18, 39; 9.19	0.001
Snout-vent length	0.87	18, 39; 6.99	0.001

**Table 5:** Comparison of traits between snake hatchlings born from unturned (top embryos) or turned eggs (bottom embryos).

Egg treatment	Unturned (top)	Turned (bottom)	df, F	P
Incubation duration (days)	44.86 ± 1.36	44.37 ± 1.45	1, 36; 1.13	0.29
Emergence time (days)	0.36 ± 0.49	0.69 ± 0.48	1, 36; 4.10	**0.050**
Residual yolk index	−0.027 ± 0.22	0.14 ± 0.17	1, 36; 0.38	0.54
Body mass (g)	2.26 ± 0.41	2.16 ± 0.49	1, 36; 0.61	0.44
Snout-vent length (cm)	13.01 ± 1.02	12.94 ± 0.99	1, 36; 0.05	0.84
Body condition index	0.0074 ± 0.049	−0.010 ± 0.042	1, 36; 1.32	0.26
Swimming speed (cm.s^-1^)	39.72 ± 8.04	42.28 ± 10.87	1, 23; 0.44	0.51
Swimming speed (body length.s^-1^)	3.02 ± 0.56	3.14 ± 0.71	1, 23; 0.19	0.67
Time to exit shelter (min)	44.45 ± 21.07	52.25 ± 19.19	1, 25; 0.87	0.36
Defensive score	0.71 ± 1.35	0.89 ± 1.69	1, 24; 0.09	0.77
